# Phylogenetic Classification at Generic Level in the Absence of Distinct Phylogenetic Patterns of Phenotypical Variation: A Case Study in Graphidaceae (Ascomycota)

**DOI:** 10.1371/journal.pone.0051392

**Published:** 2012-12-12

**Authors:** Sittiporn Parnmen, Robert Lücking, H. Thorsten Lumbsch

**Affiliations:** Botany Department, The Field Museum, Chicago, Illinois, United States of America; University of California, Berkeley, United States of America

## Abstract

Molecular phylogenies often reveal that taxa circumscribed by phenotypical characters are not monophyletic. While re-examination of phenotypical characters often identifies the presence of characters characterizing clades, there is a growing number of studies that fail to identify diagnostic characters, especially in organismal groups lacking complex morphologies. Taxonomists then can either merge the groups or split taxa into smaller entities. Due to the nature of binomial nomenclature, this decision is of special importance at the generic level. Here we propose a new approach to choose among classification alternatives using a combination of morphology-based phylogenetic binning and a multiresponse permutation procedure to test for morphological differences among clades. We illustrate the use of this method in the tribe Thelotremateae focusing on the genus *Chapsa*, a group of lichenized fungi in which our phylogenetic estimate is in conflict with traditional classification and the morphological and chemical characters do not show a clear phylogenetic pattern. We generated 75 new DNA sequences of mitochondrial SSU rDNA, nuclear LSU rDNA and the protein-coding RPB2. This data set was used to infer phylogenetic estimates using maximum likelihood and Bayesian approaches. The genus *Chapsa* was found to be polyphyletic, forming four well-supported clades, three of which clustering into one unsupported clade, and the other, supported clade forming two supported subclades. While these clades cannot be readily separated morphologically, the combined binning/multiresponse permutation procedure showed that accepting the four clades as different genera each reflects the phenotypical pattern significantly better than accepting two genera (or five genera if splitting the first clade). Another species within the Thelotremateae, *Thelotrema petractoides*, a unique taxon with carbonized excipulum resembling *Schizotrema*, was shown to fall outside *Thelotrema*. Consequently, the new genera *Astrochapsa, Crutarndina, Pseudochapsa,* and *Pseudotopeliopsis* are described here and 39 new combinations are proposed.

## Introduction

Molecular data have revolutionized our understanding of the evolution of organisms and have had profound impact on classifications, especially in organisms lacking complex morphologies, such as fungi [1]–[3]. Traditionally, the classification of living organisms has worked under the paradigm that taxa should be recognizable, i.e. having phenotypic features that delimit them from other taxa. However, a major challenge of the results of molecular phylogenetic studies is that lineages often do not correlate well with phenotypic features [4]–[11]. In these cases, re-examination of phenotypical characters often fails to identify diagnostic characters, especially in organismal groups lacking complex morphologies. Reasons for the absence of phenotypical differences among clades include convergent evolution, parallel but independent transformations of morphological characters in related lineages, as well as morphostasis and retention of ancestral features [12]–[16]. Hence, delimitation based on morphology alone can be difficult or even impossible. This problem has long been recognized and accepted at higher taxonomic levels, such as orders and families, which often cannot be circumscribed by phenotypic characters. Even so, their formal recognition does not appear to pose any conceptual problem, as apparent from widely accepted classifications [1], [17].

Due to the nature of binomial nomenclature introduced by Linnaeus, in which a species name is composed of the generic name and the epitheton, changes in the classification of an organism at the generic level lead to a change in the name of the organism. Thus, systematists have been reluctant to translate phylogenetic studies into classification at the generic level when monophyletic clades lack correlating phenotypical features. When a genus-level taxon is found to be poly- or paraphyletic, it can be either split or merged with another taxon to obtain monophyly. Since genera and all higher taxonomic ranks are arbitrary, both lumping or splitting would be possible and there is no a priori scientific argument to favor either solution. Such reclassifications often lead to genera that are not distinguishable by phenotypical characters, and these have been called “cryptic genera” [18]–[21] analogous to “cryptic species”, which are morphologically undistinguishable [22]–[28].

Here we propose a new, quantitative approach to choose among alternative classifications, combining the technique of morphology-based phylogenetic binning with a multi-response permutation procedure (MRPP) [29]–[31]. Phylogenetic binning [32] is a method that determines the level of congruence between phenotypical site patterns and molecular phylogenies and then applies character weights in order to improve the accuracy of the classification of taxa for which no molecular data are yet available. The advantage of this method is the individual placement of taxa in a reference tree based on closest relationship, rather than overall difference between clades. Thus, this method yields better results than simultaneous clustering or cladistic analysis of many taxa based on morphological data. MRPP compares average distances between groups based on characters (putative taxa), using data randomization to obtain evidence of statistical significance. Both methods can be combined to test alternative classification models in order to evaluate which classification best fits both the phylogenetic topology and the morphological data, under the criterion that resulting taxa should be monophyletic.

We used the tribe Thelotremateae in Graphidaceae, a family of lichenized fungi, to illustrate our approach [33], [34]. This clade includes four currently accepted genera: *Chapsa, Chroodiscus, Leucodecton,* and *Thelotrema.* Previous studies showed that *Chroodiscus* and *Leucodecton* are monophyletic [33]–[36], while *Chapsa* was shown to be highly polyphyletic. The core of *Thelotrema* was found to be monophyletic with one species having unclear phylogenetic reltionships. The genus *Chapsa* in its current sense is characterized morphologically by so-called chroodiscoid apothecia with widely open disc bordered by a splitting, lobulate margin, as well as the presence of lateral paraphyses, which are hyphae growing into the hymenium from the lateral margin of the fruiting body [37], [38]. However, *Chapsa* was found to consist of unrelated lineages [33] that fall both inside and outside the Thelotremateae, and even the *Chapsa* species within Thelotremateae form at least two clades, one of which having low support but including three subclades with strong support, whereas the other clade includes two supported subclades. At first glance, there are no apparent phenotypic characters that would separate these clades, since thallus morphology, ascospore type, and secondary chemistry vary widely in each clade. As a consequence, one could recognize a single genus *Thelotrema* for all Thelotremateae, which would, however, not do justice to the morphologically and phylogenetically well-defined clades representing *Chroodiscus*, *Leucodecton*, and *Thelotrema* sensu stricto. The preferred alternative would be splitting *Chapsa* into more than one genus, but without any obvious, supporting morphological characters the decision for either two, four, or even five genera would be arbitrary. Our approach provides statistical evidence that helps to choose among alternatives and we consider this a model case in how to tackle classifications of morphologically complex groups with clear underlying phylogenetic topologies.

## Results

### Phylogenetic Analyses

**Figure 1:**
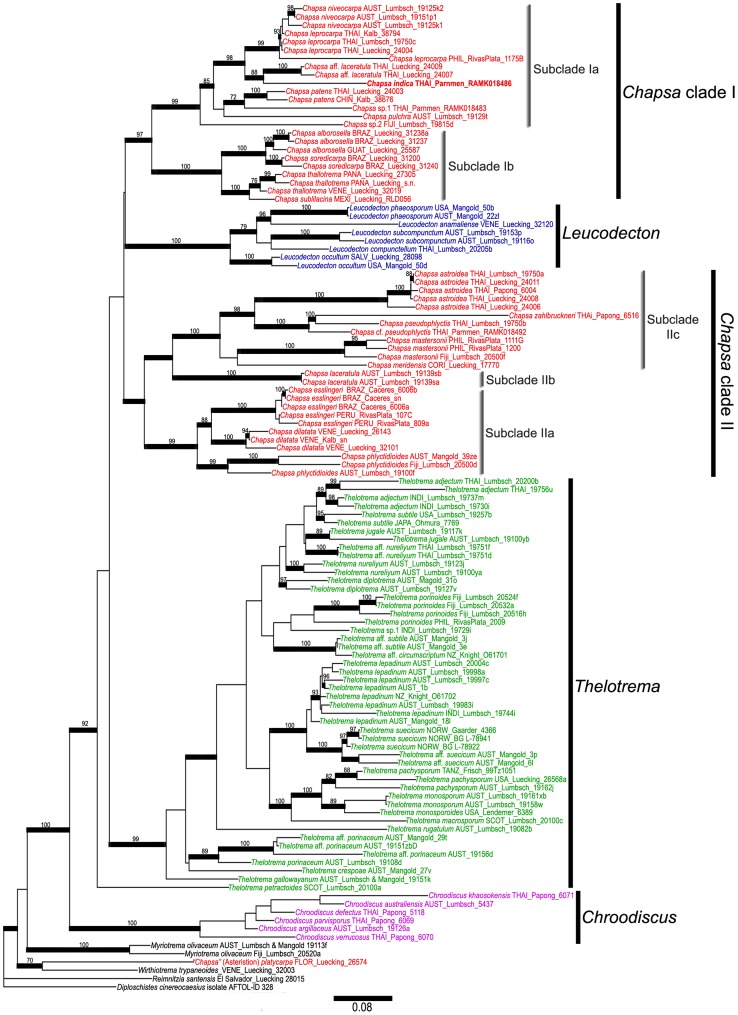
Bayesian 50% majority-rule consensus tree depicting relationships among genera in the tribe Thelotrematae on the basis of a concatenated data set including mtSSU rDNA, nuLSU rDNA and protein-coding RPB2. Posterior probabilities equal or above 0.95 are indicated as bold branches. ML-bootstrap support equal or above 70% is shown as number at branches.

Seventy-five new sequences were generated for this study and aligned with 237 sequences downloaded from Genbank, most of them generated in our lab and included in a previous study (Table 1). The combined data matrix of 2482 unambiguously aligned characters with 804 characters in the nuLSU rDNA, 800 characters in mtSSU rDNA and 878 characters in *RPB2* was used for phylogenetic analyses. The single gene analyses did not show any conflicts and hence the concatenated data set was analyzed. The ML tree had a likelihood value of –38,803.262 and in the B/MCMC analysis of the combined data set, the likelihood parameters in the sample had the following mean (Variance): LnL = –42,231.616 (0.17). The maximum likelihood tree did not contradict the Bayesian tree topologies and hence only the majority-rule consensus tree of the Bayesian tree sampling is shown (Fig. 1).

**Table 1:** Species and specimens used in the present study, with location, reference collection details, and GenBank accession numbers. Newly obtained sequenced in bold.

Species	Collection data	mtSSU acc. no.	nuLSU acc. no	*RPB2* acc. no
*Chapsa alborosella*	Brazil, Lücking 31237 (F)	JX420971	JX421438	**JX465320**
*C. alborosella*	Brazil, Lücking 31238a (F)	JX420972	JX421439	JX420936
*C. alborosella*	Guatemala, Lücking 25587 (F)	JX420973	JX421440	JX420940
*C. astroidea*	Thailand, Lumbsch 19750a (F)	JX420974	JX421441	JX420859
*C. astroidea*	Thailand, Lücking 24011 (F)	**JX465278**	JX421445	JX420947
*C. astroidea*	Thailand, Papong 6004 (F)	JX420975	JX421442	JX420865
*C. astroidea*	Thailand, Lücking 24008 (F)	JX420978	JX421444	JX420945
*C. astroidea*	Thailand, Lücking 24006 (F)	JX420977	JX421443	JX420943
*C. dilatata*	Venezuela, Kalb s.n.	JX420980	–	JX420898
*C. dilatata*	Venezuela, Lücking 32101 (F)	JX420981	JX421446	JX420906
*C. dilatata*	Venezuela, Lücking 26143 (F)	JX420982	JX421447	JX420949
*C. esslingeri*	Brazil, Cáceres 6006a	JX420984	–	JX420885
*C. esslingeri*	Brazil, Cáceres 6006b (F)	**JX465279**	–	JX420886
*C. esslingeri*	Brazil, Cáceres s.n. (F)	JX420983	–	JX420883
*C. esslingeri*	Peru, Rivas Plata 107C (F)	JX420985	–	JX420870
*C. esslingeri*	Peru, Rivas Plata 809a (F)	JX420986	**JX465294**	**JX465321**
*C. indica*	Thailand, Parnmen 018486 (RAMK)	**JX465280**	**JX465295**	**JX465322**
*C. laceratula*	Australia, Lumbsch 19139sa (F)	JX420988	JX421448	JX420831
*C. laceratula*	Australia, Lumbsch 19139sb (F)	JX420989	**JX875070**	–
*C.* aff. *laceratula*	Thailand, Lücking 24007 (F)	JX420969	JX421436	JX420944
*C.* aff. *laceratula*	Thailand, Lücking 24009 (F)	JX420970	JX421437	JX420946
*C. leprocarpa*	Thailand, Kalb 38794 (hb. Kalb)	JX420994	JX421453	JX420928
*C. leprocarpa*	Thailand, Lumbsch 19750c (F)	JX420993	JX421452	JX420857
*C. leprocarpa*	Thailand, Lücking 24004 (F)	JX420995	JX421455	JX420942
*C. leprocarpa*	Philippines, Rivas Plata 1175B (F)	JX420992	**JX465296**	**JX465323**
*C. mastersonii*	Fiji, Lumbsch 20500f (F)	JX420996	**JX465297**	**JX465324**
*C. mastersonii*	Philippines, Rivas Plata 1111G (F)	JX420998	**JX465298**	JX420860
*C. mastersonii*	Philippines, Rivas Plata 1200 (F)	JX420999	**JX465299**	JX420861
*C. meridensis*	Costa Rica, Lücking 17770 (F)	EU075610	EU075655	JF828940
*C. niveocarpa*	Australia, Lumbsch 19125k1 (F)	EU075568	EU075615	–
*C. niveocarpa*	Australia, Lumbsch 19125k2 (F)	EU675274	–	–
*C. niveocarpa*	Australia, Lumbsch 19151p1 (F)	EU075567	FJ708487	–
*C. patens*	Thailand, Lücking 24003 (F)	JX421003	JX421459	JX420941
*C. patens*	China, Kalb 38676 (hb. Kalb)	JX421001	JX421458	JX420939
*C. phlyctidioides*	Australia, Mangold 39ze (F)	EU675275	**JX465300**	**JX465326**
*C. phlyctidioides*	Fiji, Lumbsch 20500d (F)	JX421005	**JX465301**	**JX465327**
*C. phlyctidioides*	Australia, Lumbsch 19100f (F)	EU075569	**JX465302**	**JX465325**
*C. platycarpa*	USA, Lücking 26573 (F)	JX421007	JX421460	**JX465328**
*C. pseudophlyctis*	Thailand, Lumbsch 19750b (F)	JX421008	**JX465303**	JX420858
*C.* cf. *pseudophlyctis*	Thailand, Parnmen 018492 (RAMK)	**JX465277**	**JX465293**	**JX465331**
*C. pulchra*	Australia, Lumbsch 19129t (F)	EU075571	EU075619	**JX465329**
*C. soredicarpa*	Brazil, Lücking 31200 (F)	JX421011	JX421462	JX420935
*C. soredicarpa*	Brazil, Lücking 31240 (F)	JX421012	JX421463	JX420937
*C. sublilacina*	Mexico, Lücking RLD056 (F)	HQ639600	JX421466	JX420842
*C. thallotrema*	Venezuela, Lücking 32019 (F)	JX421013	**JX467681**	JX420905
*C. thallotrema*	Panama, Lücking 27305 (F)	**JX465282**	**JX465305**	**JX465333**
*C. thallotrema*	Panama, Lücking s.n. (F)	**JX465283**	**JX465306**	**JX465334**
*Chapsa* sp.1	Thailand, Parnmen 018483 (RAMK)	**JX465281**	**JX465304**	**JX465332**
*Chapsa* sp.2	Fiji, Lumbsch 19815d (F)	JX421000	JX421457	**JX465330**
*Chroodiscus argillaceus*	Australia, Lumbsch 19126a (F)	HQ639585	JX421468	–
*C. australiensis*	Australia, Lumbsch 5437 (F)	FJ708496	FJ708489	–
*C. defectus*	Thailand, Papong 5118 (F)	FJ708497	FJ708490	–
*C. khaolungensis*	Thailand, Papong 6071 (F)	**JX465284**	–	**JX465335**
*C. parvisporus*	Thailand, Papong 6069 (F)	**JX465285**	JX421469	JX420863
*C. verrucosus*	Thailand, Papong 6070 (F)	**JX465286**	**JX465307**	–
*Diploschistes cinereocaesius*	AFTOL-ID 328	DQ912306	DQ883799	DQ883755
*Leucodecton anamaliense*	Venezuela, Lücking 32120 (F)	JX421077	JX421512	**JX465336**
*L. compunctellum*	Thailand, Lumbsch 20205b (F)	JX421081	JX421514	–
*L. occultum*	El Salvador, Lücking 28098 (F)	HQ639611	HQ639657	JF828949
*L. occultum*	USA, Mangold 50d (F)	JX421084	–	JX420846
*L. phaeosporum*	Australia, Mangold 22zl (F)	JF828962	–	–
*L. phaeosporum*	USA, Mangold 50a (F)	**JX465287**	**JX465308**	**JX465337**
*L. subcompunctum*	Australia, Lumbsch 19153p (F)	EU075576	EU075624	–
*L. subcompunctum*	Australia, Lumbsch 19116o (F)	EU075575	EU075623	–
*Myriotrema olivaceum*	Australia, Lumbsch & Mangold 19113f (F)	EU075579	EU075627	HM244799
*M. olivaceum*	Fiji, Lumbsch 20520a (F)	**JX465288**	JX421531	**JX465338**
*Reimnitzia santensis*	El Salvador, Lücking 28015 (F)	HQ639622	–	JF828952
*Thelotrema adjectum*	India, Lumbsch 19730i (F)	JX421344	JX421642	JX420851
*T. adjectum*	India, Lumbsch 19737m (F)	JX421343	JX421641	JX420848
*T. adjectum*	Thailand, Lumbsch 20200b (F)	JX421347	JX421645	**JX465350**
*T. adjectum*	Thailand, Lumbsch 19756u (F)	**JX465289**	JX421644	JX420853
*T.* aff. *circumscriptum*	New Zealand, Knight 61701 (F)	**JX465290**	–	**JX465339**
*T. crespoae*	Australia, Mangold 27v (F)	DQ384917	FJ708493	–
*T. diplotrema*	Australia, Mangold 31o (F)	JX421356	–	JX420847
*T. diplotrema*	Australia, Lumbsch 19127v (F)	EU075599	JX421649	JX420827
*T. gallowayanum*	Australia, Lumbsch 19151k (F)	EU075600	EU075653	–
*T. jugale*	Australia, Lumbsch 19117k (F)	EU675293	–	–
*T. jugale*	Australia, Lumbsch 19100yb (F)	JX421360	**JX465310**	JX420826
*T. lepadinum*	Australia, Lumbsch 20004c (F)	JX421370	JX421655	JX420868
*T. lepadinum*	Australia, Lumbsch 19998a (F)	JX421368	**JX465311**	JX420866
*T. lepadinum*	Australia, Lumbsch 19997c (F)	JX421369	JX421654	JX420867
*T. lepadinum*	Australia, Mangold 1b (F)	JX421362	JX421656	JX420837
*T. lepadinum*	Australia, Mangold 18l (F)	JX421363	JX421651	JX420840
*T. lepadinum*	Australia, Lumbsch 19983i (F)	JX421371	**JX465312**	–
*T. lepadinum*	India, Lumbsch 19744i (F)	JX421365	JX421652	JX420850
*T. lepadinum*	New Zealand, Knight 61702 (F)	JX421366	JX421653	JX420934
*T. macrosporum*	UK, Scotland, Lumbsch 20100c (F)	**JX465291**	**JX465313**	JX420890
*T. monosporoides*	USA, Lendemer 6389 (NY)	EU075602	EU075645	**JX465340**
*T. monosporum*	Australia, Lumbsch 19161xb (F)	EU075596	EU075644	–
*T. monosporum*	Australia, Lumbsch 19158w (F)	EU075601	EU075646	–
*T. nureliyum*	Australia, Lumbsch 19123j (F)	JX421376	JX421660	**JX465341**
*T. nureliyum*	Australia, Lumbsch 19100ya (F)	EU075597	EU075647	–
*T.* cf. *nureliyum*	Thailand, Lumbsch 19751f (F)	JX421408	JX421673	JX420854
*T.* cf. *nureliyum*	Thailand, Lumbsch 19751d (F)	JX421409	JX421674	JX420856
*T. pachysporum*	Tanzania, Frisch 99Tz1051 (hb. Kalb)	DQ384918	DQ431925	–
*T. pachysporum*	USA, Lücking 26568a (F)	JX421381	–	**JX465342**
*T. pachysporum*	Australia, Lumbsch 19162j (F)	EU675290	–	JX420829
*T. petractoides*	UK, Scotland, Lumbsch 20100a (F)	JX421383	JX421664	JX420891
*T. porinaceum*	Australia, Lumbsch 19108d (F)	JX421384	**JX465314**	**JX465343**
*T.* aff. *porinaceum*	Australia, Mangold 29t (F)	JX421350	JX421646	JX420844
*T.* aff. *porinaceum*	Australia, Lumbsch 19151zb (F)	JX421349	–	**JX465344**
*T.* aff. *porinaceum*	Australia, Lumbsch 19156d (F)	EU675291	**JX465309**	–
*T. porinoides*	Fiji, Lumbsch 20524f (F)	JX421394	–	**JX465346**
*T. porinoides*	Fiji, Lumbsch 20532a (F)	JX421397	**JX465315**	**JX465347**
*T. porinoides*	Fiji, Lumbsch 20516h (F)	JX421386	**JX465316**	**JX465345**
*T. porinoides*	Philippines, Rivas Plata 2009 (F)	HQ639603	JX421665	–
*T. rugatulum*	Australia, Lumbsch 19082b (F)	EU075605	**JX465317**	–
*T. subtile*	USA, Lumbsch 19257b (F)	JX421402	–	JX420836
*T. subtile*	Japan, Ohmura 7769 (TNS)	JX421403	JX421668	JX420932
*T.* aff. *subtile*	Australia, Mangold 3e (F)	EU675297	DQ871013	**JX465348**
*T.* aff. *subtile*	Australia, Mangold 3j (F)	EU075607	EU075651	JX420834
*T. suecicum*	Norway, Gaarder 4365 (BG)	JX421406	JX421671	–
*T. suecicum*	Norway, Gaarder 4366a (BG)	JX421407	JX421672	JX420832
*T. suecicum*	Norway, Gaarder 4366b (BG)	**JX465292**	**JX465318**	**JX465349**
*T.* aff. *suecicum*	Australia, Mangold Am3p (F)	JX421404	JX421669	JX420833
*T.* aff. *suecicum*	Australia, Mangold Am6l (F)	JX421405	JX421670	JX420835
*Thelotrema* sp.1	India, Lumbsch 19729i (F)	JX421400	JX421667	JX420849
*Wirthiotrema trypaneoides*	Venezuela, Lücking 32003 (F)	JX421422	JX421681	JX420916

In the phylogenetic tree, the genus *Chapsa* is polyphyletic, separating into two major clades, one of which is unsupported but consists of three well-supported subclades. Distantly related species of *Chapsa* sensu lato also appear in other clades, such as *C. platycarpa* in the outgroup close to the genus *Wirthiotrema*. *Chapsa* clade I is a well-supported clade; it contains the type species, *C. indica*, and the morphologically similar *C. leprocarpa*, *C. niveocarpa*, *C. patens*, and *C. pulchra*, but also *C. alborosella* and the morphologically quite disparate *C. sublilacina* and relatives. The clade forms two supported subclades, one containing *C. alborosella* and *C. sublilacina*, among other species, and the other *C. indica*, *C. leprocarpa*, and *C. patens*, among other species. *Chapsa* clade II can be divided into subclades IIa, IIb, and IIc. Subclade IIa contains *C. dilatata* and *C. phlyctidioides*, which morphologically resemble species of Clade I; subclade IIb comprises the single species *C. laceratula*, which resembles a *Topeliopsis* in apothecial morphology but with well-developed, corticate thallus; and subclade IIc includes the morphologically disparate *C. astroidea*, *C. mastersonii*, and *C. zahlbruckneri*, among other species. Hypothesis testing using both the SH and ELW strongly rejected the monophyly of *Chapsa*, even if only considering the species falling within the Thelotremateae (*p*≤0.0001 in both tests). The *Thelotrema* clade is supported as a monophyletic group, but excluding *Thelotrema petractoides,* which falls outside the main clade as an early diverging taxon with uncertain phylogenetic relationships.

### Phylogenetic Binning and Multi-response Permutation Procedure

Phylogenetic binning of the 65 described *Chapsa* species for which no molecular data are available suggests placement of 14 species within Clade I (*Chapsa* sensu stricto) and 49 species within Clade II under a 2-clade solution with ML weighting. Two species, *C. chionostoma* and *C. microspora*, are suggested to not form part of tribe Thelotremateae (Table 2; Appendix S1). MP weighting places 19 species in Clade I and 44 species in Clade II, suggesting again *C. chionostoma* and also *C. halei* as not belonging in tribe Thelotremateae. Between ML and MP weighting, the placement of 12 out of 65 species (19%) is conflictive (Table 2; Appendix S1). Using a 4-clade solution, ML weighting places 16 out of 65 species in Clade I, 24 species in Clade IIa, five species in Clade IIb, and 20 species in Clade IIc; in contrast, MP weighting places 21 out of 65 species in Clade I, 21 species in Clade IIa, seven species in Clade IIb, and 16 species in Clade IIc. The placement of 19 species (29%) is conflictive between ML and MP weighting. Under a 5-clade solution, splitting Clade I into subclades Ia (Chapsa s.str.) and Ib (*alborosella*-*sublilacina* clade), of the 16 species placed in Clade I, eight fall into Clade Ib with ML weighting and 11 with MP weighting. Six of these are identical whereas seven are conflictive between the two weighting techniques, for a total of 20 conflictive placements in the 5-clade solution (Table 2).

**Table 2:** Placement of species within clades based on morphological characters using phylogenetic binning method under ML and MP weighting.

	ML 2-clades	MP 2-clades	ML 4-clades	MP 4-clades	ML 5-clades	MP 5-clades
Clade I	14	19	16	21	–	–
Subclade Ia	–	–	–	–	8	10
Subclade Ib	–	–	–	–	8	11
Clade II	49	44	–	–	–	–
Subclade IIa	–	–	24	21	24	21
Subclade IIb	–	–	5	7	5	7
Subclade IIc	–	–	20	16	20	16
Outside	2	2	0	0	0	0
Conflicting	12		19		20	
**Total**	**65**	**65**	**65**	**65**	**65**	**65**

The MRPP analysis resulted in non-significant or spuriously significant differences between groups for the 2-clade solution but in highly significant differences for the 4-clade solution, independent of group assignment based on ML or MP weighting and of the distance measure employed (Table 3). Group assignments based on MP weighting gave slightly better correlations than based on ML weighting, as did the Euclidean distance measure compared to a linear correlation coefficient (Table 3). This suggests that the 4-clade solution and group assignment based on MP weighting is the best fit to the data.

**Table 3:** Results of the multi-response permutation procedure (MRPP) analysis.

	Euclidean	Correlation
ML 2-clades	0.0732	0.1697
MP 2-clades	0.0366	0.0785
ML 4-clades	0.0000	0.0000
MP 4-clades	0.0000	0.0000
ML 5-clades	0.0000	0.0000
MP 5-clades	0.0000	0.0000

p-values for significance of group distances based on morphological character matrix.

Kruskal-Wallis ANOVA indicates five characters as significantly discriminating between groups in a 2-clade solution using ML weighting and an additional three characters as marginally significant (Table 4). MP weighting results in a similar pattern but with overall fewer discriminating characters. Both the 4-clade and the 5-clade solutions suggests a much higher number of discriminating characters, again with a higher total for ML weighting. This supports the 4-clade or 5-clade solutions providing a better fit to the data than the 2-clade solution, with a slight advantage for ML over MP weighting.

**Table 4:** Number of individual characters discriminating between groups using phylogenetic binning method under ML and MP weighting.

	Significant (p<0.05)	Marginally significant (p<0.10)	Total
ML 2-clades	5	3	8
MP 2-clades	3	3	6
ML 4-clades	15	3	18
MP 4-clades	11	3	14
ML 5-clades	14	4	18
MP 5-clades	11	3	14

The best discriminating characters in the 4-clade and 5-clade solutions (Table 5) are the presence of soralia (MP weighting only), the nature of the thallus cortex, ascoma exposure and the shape of the proper and thalline margin, excipulum carbonization, ascospore number and dimensions (ML weighting only), ascospore endospore development and iodine reaction (best discriminating character under both ML and MP weighting), ascospore septation, and secondary chemistry (stictic and protocetraric acid; ML weighting only). According to these results, species of Clades Ia and IIa tend to have a loose cortex or lack a cortex altogether, whereas species of Clades Ib, IIb and IIc mostly have a dense cortex. Soralia are entirely confined to Clade I and particularly Clade Ib. Brown excipula are significantly more frequent in Clades IIa and IIc. Ascospores with amyloid endospore are particularly frequent in Clades Ib and IIa, and the latter clade also tends to have ascospores with a lower number of transverse septa than the other clades. The partial differences found in the level of character discrimination between taxon placement based on ML or MP weighting correlate with the weights determined for each character in the phylogenetic binning analysis. Characters that received high weights under an ML approach but low weights under MP include the thallus cortex, ascospore number and dimensions, and secondary chemistry, whereas the opposite was found for characters such as the presence of soralia.

**Table 5:** Discriminating characters as based on a Kruskal-Wallis ANOVA using the different clade solutions as grouping variables. P-values are indicated if below 0.1.

	ML-2	MP-2	ML-4	MP-4	ML-5	MP-5
Soralia		0.0019		0.0102		0.0002
Oxalate crystals				0.0129		0.0244
Cortex			0.0744	0.0914	0.0089	0.0313
Ascoma emergence				0.0296		0.0567
Ascoma shape					0.0962	0.0504
Ascoma aggregation	0.0975		0.0544			
Ascoma diameter	0.0661	0.0980	0.0574		0.0596	
Ascoma exposure			0.0017	0.0010	0.0043	0.0026
Proper margin shape			0.0368	0.0168		0.0057
Proper margin striation			0.0262	0.0019	0.0741	0.0049
Proper margin split			0.0007	0.0000	0.0544	0.0000
Thallus margin shape				0.0003	0.0003	0.0002
Excipulum carbonization	0.0080	0.0148	0.0123	0.0391	0.0274	0.0592
Periphysoids presence	0.0000	0.0000				
Ascospores number			0.0033		0.0080	
Ascospores length	0.0037	0.0723	0.0003		0.0008	
Ascospores width	0.0773	0.0862	0.0077		0.0168	
Ascospores length-to-widthratio	0.0006		0.0014		0.0027	
Ascospores endosporedevelopment			0.0000	0.0000	0.0000	0.0000
Ascospores iodine reaction			0.0000	0.0000	0.0000	0.0000
Ascospores transverse septa	0.0056		0.0003	0.0919	0.0008	
Ascospores longitudinal septa			0.0002	0.0546	0.0005	0.0272
Chemistry stictic acid			0.0366		0.0405	
Chemistry protocetraric acid			0.0005		0.0010	

## Discussion

The detection of phylogenetically defined clades lacking clearly discriminant morphological characters is not rare and particularly common in fungal groups, including lichenized species, since these organisms are composed of rather simple structures and are less differentiated than higher plants and animals [39], [40]. At higher taxonomic levels, such as family and order, this phenomenon has already found broad acceptance in fungal classifications [1], [17], [41]. Also, increasing evidence points to the frequent occurrence of cryptic species [22], [23], [25], [27], [28]. At the generic level, however, systematists have been highly reluctant to accept so-called cryptic genera, mainly because in lichenized fungi, the genus level has been the main taxonomic entity for classification purposes and herbaria collections are mostly organized using this taxonomic category. It is also commonly expected that classifications should result in the recognition of taxa, particularly at the genus level, that are phenotypically recognizable. However, increasing evidence from phylogenetic studies indicates that, while many monophyletically circumscribed genera are indeed recognizable, in other cases clades are inconsistent with morphological data. In many cases, particular morphotypes form either paraphyletic grades, with other morphotypes nested within, or are polyphyletic. Since the objective of molecular phylogenetic studies is to recognize natural groups, such para- or polyphyletic taxa cannot be maintained, except in the case of recently evolved species that have experienced the founder effect [42]–[45].

The genus *Chapsa* [37] had already been suspected to be not monophyletic, but the split into up to five clades within tribe Thelotremateae, and the placement of additional species outside this tribe, poses a challenge to classification, since there are no straightforward characters or a combination thereof that can be used to distinguish these lineages phenotypically. This situation is not rare in Graphidaceae and has also been found in the genera *Graphis* versus *Allographa*, *Myriotrema* versus *Ocellis*, *Leucodecton* versus *Wirthiotrema*, and *Leucodecton* versus *Leptotrema*, versus *Ocellularia* [15], [32], [33], [46]. There are numerous other examples of this situation among fungi and they are usually accepted if the lineages are unrelated or distantly related, but disputed in case of closer relationships, even if the underlying problem is the same. Thus, the basidiolichen genera *Multiclavula* and *Lepidostroma* include species that cannot be separated by any phenotypical character, but their very distant position among the Basidiomycota provides grounds for their taxonomic separation [47], [48]. The opposite phenomenon is also not rare: closely related lineages that are widely disparate morphologically, such as the genera *Cruentotrema* and *Dyplolabia* in Graphidaceae [15]. It is surprising that morphologically variable lineages merged into a single taxon are more readily accepted than separate, morphologically cryptic lineages, even if the underlying problem of lack of phenotypic consistency is the same.

In the case of the genus *Chapsa*, the situation is especially complex since up to five clades can be distinguished based on molecular phylogeny in the tribe Thelotremateae alone. Our approach shows that a 4-clade or 5-clade solution fits the data much better that a 2-clade solution, since the between-group differences are highly significant and the number of discriminating characters is much higher than in a 2-clade solution. The data do not allow to determine whether the 4-clade or 5-clade solution is the best fit (except that the latter has a slightly higher number of conflictive placements), since both are highly significant in terms of morphological discrimination and have about the same number of discriminating characters. Because of the lack of difference between the two alternatives, and since the branch leading to Clade I has high support, we opt for the more conservative solution here and maintain Clade I as a single genus, *Chapsa*. Our results suggest that there is a strong tendency for each of the four clades to differ in thallus cortex type, excipular carbonization, and ascospore type and septation (especially endospore development and iodine reaction), which is consistent with each clade having a long stem node and hence having evolved internal morphological variation independently. However, each clade includes a few species that morphologically would better fit in another clade. Such cases must be interpreted as either ancestral characters retained in a clade or as examples of parallel evolution. For this reason, these characters, even if statistically discriminant between genus-level clades, cannot be used to actually key out the genus-level taxa themselves.

The phylogenetic binning method allows both ML and MP weighting of the morphological characters, but the underlying algorithms are different [32]. For ML weighting, the best-scoring molecular tree is compared to a set of randomized trees (e.g. 100 trees) and the weight is derived by the number of random trees in which a particular morphological character mapped on the tree receives a worse log likelihood score. If a character has a strongly consistent distribution on the best-scoring molecular tree, any randomized tree will have a lower log likelihood score for this character, and the weight will be 100%. In contrast, MP weighting is derived only from the best-scoring molecular tree, and parsimony scores are computed by mapping the morphological characters on the tree and converting the scores into weights. As a result, ML weighting will emphasize characters that are confined to particular major clades (absolute synapomorphies), whereas MP weighting will also give higher weights to characters that characterize more than one clade but are absent from others (relative synapomorphies). Which method works better depends on the context, but the slightly better MRPP results for MP weighting in the 2-clade solution in this study confirm the findings of the original paper [32] that MP weighting might give slightly more consistent results. Interestingly, the characters found here to receive higher weights under ML are ascospore number and dimensions as well as number of septa, whereas under MP their weight was zero. These characters are known to vary strongly even within clades but are usually consistent between more closely related species, which could cause the effect that randomized trees consistently give lower log likelihood scores even if the overall character distribution over the tree is near-random. It is therefore recommended to use both ML and MP weighting in combination and closely inspect taxa with conflicting placement under both approaches, but if both methods yield quantitatively similar results overall, the MP solution is preferable as done here.

In contrast to making phenotypical characters obsolete in systematics, our study underlines the importance of these data even in times where molecular data become increasingly available to reconstruct phylogenies. While phenotypical data itself should not be used in such reconstructions, they are indispensable when transforming phylogenies into classifications and, with powerful analytical methods, provide statistical evidence that can be used to compare alternative classification models based on an underlying phylogeny.

### Taxonomic Conclusions

The results of our phylogenetic analysis and the combined binning/multiresponse permutation procedure support a classification accepting each of the four Chapsa clades as different genera. Also, *Thelotrema petractoides* was shown to be distantly related to the core genus. Consequently, the new genera *Astrochapsa, Crutarndina, Pseudochapsa*, and *Pseudotopeliopsis* are described below and 39 new combinations are proposed. We only propose new combinations for species without conflict regarding clade placement under either ML or MP weighting, whereas the conflictive species are provisionally retained in *Chapsa* until sequence data become available. Therefore, the number or proposed combinations is lower than the numbers indicated in Table 2 for each clade. For example, the results suggest to place 24 taxa under ML and 21 taxa under MP weighting in *Pseudochapsa*, but only 16 of these are identical with both approaches, and only these are recombined here. In addition, we refrained from formally recombining five taxa that did not exhibit conflict but are suspected to possibly fall outside the Thelotremateae and hence require sequencing to clarify their position. This might also apply to some of the taxa with conflicting placement, such as *C. asteliae* and *C. lordhowensis* (see Appendix S1). Two species were recombined in *Pseudotopelipsis* favoring the MP weighting solution.

#### Astrochapsa

Parnmen, Lücking & Lumbsch, gen. nov. [MycoBank MB 801540] Type species: *Astrochapsa astroidea* (Berk. & Broome) Parnmen, Lücking & Lumbsch.

Differing from *Chapsa* s.str. in the more frequently densely corticate thallus, the mostly recurved apothecial margin, and the almost exclusively subdistoseptate, non-amyloid ascospores.

Thallus usually with dense cortex, rarely with loose cortex or ecorticate. Apothecia erumpent, rounded to irregular in outline; disc exposed; margin lobulate to usually recurved. Excipulum usually brown. Ascospores septate to muriform, fusiform-ellipsoid to oblong-cylindrical, with slightly thickened septa and angular lumina (subdistoseptate), colorless or rarely brown, almost exclusively I–. Secondary chemistry: no substances or frequently stictic acid and relatives; apothecial disc sometimes pigmented.

Etymology: Derived from “astro” (Greek, starry) because of the star-like morphology of the ascomata and the genus name *Chapsa.*

### New Combinations in *Astrochapsa*

#### Astrochapsa alstrupii

(Frisch) Parnmen, Lücking & Lumbsch, comb. nov. [MycoBank MB 801500] Bas.: *Chapsa alstrupii* Frisch, Biblioth. Lichenol. 92: 93 (2006).

#### Astrochapsa amazonica

(Kalb) Parnmen, Lücking & Lumbsch, comb. nov. [MycoBank MB 801501] Bas.: *Chapsa amazonica* Kalb, Herzogia 22: 24 (2009).

#### Astrochapsa astroidea

(Berk. & Broome) Parnmen, Lücking & Lumbsch, comb. nov. [MycoBank MB 801502] Bas.: *Platygrapha astroidea* Berk. & Broome, J. Linn. Soc., Bot. 14: 109 (1873); *Chapsa astroidea* (Berk. & Broome) Cáceres & Lücking in Cáceres, Libri Botanici 22: 51 (2007).

#### Astrochapsa calathiformis

(Vain.) Parnmen, Lücking & Lumbsch, comb. nov. [MycoBank MB 801503] Bas.: *Thelotrema calathiforme* Vain., Hedwigia 46: 174 (1907); *Chapsa calathiformis* (Vain.) Lumbsch & Papong in Papong et al., Lichenologist 42: 136 (2010).

#### Astrochapsa graphidioides

(Kalb) Parnmen, Lücking & Lumbsch, comb. nov. [MycoBank MB 801504] Bas.: *Chapsa graphidioides* Kalb, Herzogia 22: 24 (2009).

#### Astrochapsa lassae

(Mangold) Parnmen, Lücking & Lumbsch, comb. nov. [MycoBank MB 801505] Bas.: *Chapsa lassae* Mangold in Mangold et al., Fl. Australia 57: 653 (2009).

#### Astrochapsa magnifica

(Berk. & Broome) Parnmen, Lücking & Lumbsch, comb. nov. [MycoBank MB 801506] Bas.: *Platygrapha magnifica* Berk. & Broome, J. Linn. Soc., Bot. 14: 110 (1873); *Chapsa magnifica* (Berk. & Broome) Rivas Plata & Mangold et al., Lichenologist 42: 183 (2010).

#### Astrochapsa mastersonii

(Rivas Plata, Lumbsch & Lücking) Parnmen, Lücking & Lumbsch, comb. nov. [MycoBank MB 801507] Bas.: *Chapsa mastersonii* Rivas Plata, Lumbsch & Lücking in Weerakoon et al., Lichenologist 44: 374 (2012).

#### Astrochapsa megaphlyctidioides

(Mangold) Parnmen, Lücking & Lumbsch, comb. nov. [MycoBank MB 801508] Bas.: *Chapsa megaphlyctidioides* Mangold in Mangold et al., Fl. Australia 57: 654 (2009).

#### Astrochapsa meridensis

(Kalb & Frisch) Parnmen, Lücking & Lumbsch, comb. nov. [MycoBank MB 801509] Bas.: *Topeliopsis meridensis* Kalb & Frisch in Frisch & Kalb, Lichenologist 38: 42 (2006); *Chapsa meridensis* (Kalb & Frisch) Lücking, Lumbsch & Rivas Plata in Rivas Plata et al., Lichenologist 42: 183 (2010).

#### Astrochapsa platycarpella

(Vain.) Parnmen, Lücking & Lumbsch, comb. nov. [MycoBank MB 801510] Bas.: *Thelotrema platycarpellum* Vain., Proc. Amer. Acad. Arts Sci. 58: 138 (1923); *Chapsa platycarpella* (Vain.) Frisch, Biblioth. Lichenol. 92: 118 (2006).

#### Astrochapsa pseudophlyctis

(Nyl.) Parnmen, Lücking & Lumbsch, comb. nov. [MycoBank MB 801511] Bas.: *Graphis pseudophlyctis* Nyl. in Hue, Nouv. Arch. Mus. Nat. Hist., Sér. 3, 3: 163 (1891); *Chapsa pseudophlyctis* (Nyl.) Frisch, Biblioth. Lichenol. 92: 120 (2006).

#### Astrochapsa pulvereodiscus

(Hale) Parnmen, Lücking & Lumbsch, comb. nov. [MycoBank MB 801512] Bas.: *Thelotrema pulvereodiscus* Hale, Bull. Br. Mus. Nat. Hist., Bot. 8(3): 268 (1981); *Chapsa pulvereodiscus* (Hale) Rivas Plata & Mangold in Rivas Plata et al., Lichenologist 42: 183 (2010).

#### Astrochapsa recurva

(G. Salisb.) Parnmen, Lücking & Lumbsch, comb. nov. [MycoBank MB 801513] Bas.: *Thelotrema recurvum* G. Salisb., Rev. Bryol. Lichénol. 38: 285 (1972); *Chapsa recurva* (G. Salisb.) Frisch, Biblioth. Lichenol. 95: 120 (2006).

#### Astrochapsa stellata

**(**Hale) Parnmen, Lücking & Lumbsch, comb. nov. [MycoBank MB 801514] Bas.: *Leptotrema stellatum* Hale, Smithson. Contr. Bot. 38: 54 (1978); *Chapsa stellata* (Hale) Sipman in Sipman et al., Phytotaxa 55: 47 (2012).

#### Astrochapsa waasii

(Hale) Parnmen, Lücking & Lumbsch, comb. nov. [MycoBank MB 801515] Bas.: *Thelotrema waasii* Hale, Bull. Br. Mus. Nat. Hist., Bot. 8(3): 270 (1981); *Chapsa waasii* (Hale) Sipman & Lücking in Rivas Plata et al., Lichenologist 42: 183 (2010).

#### Astrochapsa wolseleyana

(Weerakoon, Lumbsch & Lücking) Parnmen, Lücking & Lumbsch, comb. nov. [MycoBank MB 801516] Bas.: *Chapsa wolseleyana* Weerakoon, Lumbsch & Lücking in Weerakoon et al., Lichenologist 44: 377 (2012).

#### Astrochapsa zahlbruckneri

(Redinger) Parnmen, Lücking & Lumbsch, comb. nov. [MycoBank MB 801517] Bas.: *Phaeographina zahlbruckneri* Redinger, Ark. Bot. 26A(1): 93 (1934); *Chapsa zahlbruckneri* (Redinger) Frisch, Biblioth. Lichenol. 92: 123 (2006).

#### Crutarndina

Parnmen, Lücking & Lumbsch, gen. nov. [MycoBank MB 801541] Type species: *Crutarndina petractoides* (P.M. Jørg. & Brodo) Parnmen, Lücking & Lumbsch.

Differing from *Thelotrema* s.str. in having a star-like, multi-layered exciple.

Thallus ecorticate. Apothecia erumpent, rounded; disc largely obscured by exciple; margin star-like, multi-layered. Excipulum hyaline basally, carbonized apically. Ascospores transversaly septate, fusiform, with thickened septa and lens-shaped to rounded lumina (distoseptate), colorless, I+. Secondary chemistry: no substances.

Etymology: Named after the distinguished British lichenologist Peter Crittenden (Nottingham) with whom SP and HTL collected material of the genus sequenced here on a field trip organized by the British Lichen Society. The name Crittenden is derived from the old British and Welsh and means “the cot on the lower hill”; derived from “cru” (cot); “tarn” (lower), and “dun” or “din” (hill).

### New Combination in *Crutarndina*

#### Crutarndina petractoides

(P.M. Jørg. & Brodo) Parnmen, Lücking & Lumbsch, comb. nov. [MycoBank MB 801518] Bas.: *Thelotrema petractoides* P.M. Jørg. & Brodo, in Purvis et al., Biblioth. Lichenol. 58: 352 (1995).

#### Pseudochapsa

Parnmen, Lücking & Lumbsch, gen. nov. [MycoBank MB 801542]. Type species: *Pseudochapsa dilatata* (Müll. Arg.) Parnmen, Lücking & Lumbsch.

Differing from *Chapsa* s.str. in the usually brown excipulum and the almost exclusively distoseptate, amyloid ascospores.

Thallus usually with loose cortex or ecorticate, very rarely with dense cortex. Apothecia erumpent, rounded to irregular in outline; disc exposed; margin usually fissured to lobulate, rarely recurved. Excipulum usually brown. Ascospores septate to muriform, fusiform-ellipsoid to oblong-cylindrical, mostly with thickened septa and lens-shaped to rounded lumina (distoseptate), colorless or very rarely brown, almost exclusively I+ violet-blue (amyloid). Secondary chemistry: no substances or frequently stictic acid and relatives; apothecial disc rarely pigmented.

Etymology: Derived from “pseudo” (Greek, false) and the genus name *Chapsa*.

### New Combinations in *Pseudochapsa*

#### Pseudochapsa albomaculata

(Sipman) Parnmen, Lücking & Lumbsch, comb. nov. [MycoBank MB 801519] Bas.: *Thelotrema albomaculatum* Sipman, Trop. Bryol. 5: 89 (1992); *Chapsa albomaculata* (Sipman) Sipman & Lücking in Rivas Plata et al., Lichenologist 42: 183 (2010).

#### Pseudochapsa crispata (Müll. Arg.)

Parnmen, Lücking & Lumbsch, comb. nov. [MycoBank MB 801520] Bas.: *Ocellularia crispata* Müll. Arg., J. Linn. Soc. London 30: 452 (1895); *Chapsa crispata* (Müll. Arg.) Mangold in Mangold et al., Fl. Australia 57: 653 (2009) [non *Chapsa crispata* (Müll. Arg.) Rivas Plata & Mangold in Rivas Plata et al., Lichenologist 42: 182 (2010); comb. superfl.].

#### Pseudochapsa dilatata (Kalb)

Parnmen, Lücking & Lumbsch, comb. nov. [MycoBank MB 801521] Bas.: *Ocellularia dilatata* Müll. Arg., J. Linn. Soc., London 30: 452 (1895); *Chapsa dilatata* (Müll. Arg.) Kalb, Biblioth. Lichenol. 99: 140 (2009).

#### Pseudochapsa esslingeri (Hale)

Parnmen, Lücking & Lumbsch, comb. nov. [MycoBank MB 801522] Bas.: *Ocellularia esslingeri* Hale, Smithson. Contr. Bot. 38: 20 (1978); *Chapsa esslingeri* (Hale) Sipman in Sipman et al., Phytotaxa 55: 36 (2012).

#### Pseudochapsa hypoconstictica

(Rivas Plata & Lücking) Parnmen, Lücking & Lumbsch, comb. nov. [MycoBank MB 801523] Bas.: *Chapsa hypoconstictica* Rivas Plata & Lücking, Fung. Div. (in press).

#### Pseudochapsa isidiifera

(Frisch & Kalb) Parnmen, Lücking & Lumbsch, comb. nov. [MycoBank MB 801524] Bas.: *Chapsa isidiifera* Frisch & Kalb, Biblioth. Lichenol. 99: 136 (2009).

#### Pseudochapsa kalbii

(Frisch) Parnmen, Lücking & Lumbsch, comb. nov. [MycoBank MB 801525] Bas.: *Chapsa kalbii* Frisch, Biblioth. Lichenol. 92: 103 (2006).

#### Pseudochapsa lueckingii

(Kalb) Parnmen, Lücking & Lumbsch, comb. nov. [MycoBank MB 801526] Bas.: *Chapsa lueckingii* Kalb, Herzogia 22: 25 (2009).

#### Pseudochapsa phlyctidea

(Nyl.) Parnmen, Lücking & Lumbsch, comb. nov. [MycoBank MB 801527] Bas.: *Thelotrema phlyctideum* Nyl., Ann. Sci. Nat., Bot., Sér. 4, 11: 222 (1859).

#### Pseudochapsa phlyctidioides

(Müll. Arg.) Parnmen, Lücking & Lumbsch, comb. nov. [MycoBank MB 801528] Bas.: *Ocellularia phlyctidioides* Müll. Arg., Hedwigia 32: 130 (1893); *Chapsa phlyctidioides* (Müll. Arg.) Mangold, Aust. Syst. Bot. 21: 221 (2008).

#### Pseudochapsa pseudoexanthismocarpa

(Patw. & C. R. Kulk.) Parnmen, Lücking & Lumbsch, comb. nov. [MycoBank MB 801529] Bas.: *Ocellularia pseudoexanthismocarpa* Patw. & C.R. Kulk., Norw. J. Bot. 24: 130 (1977); *Chapsa pseudoexanthismocarpa* (Patw. & C. R. Kulk.) Rivas Plata & Lücking in Rivas Plata et al., Lichenologist 42: 183 (2010).

#### Pseudochapsa pseudoschizostoma

(Hale) Parnmen, Lücking & Lumbsch, comb. nov. [MycoBank MB 801530] Bas.: *Ocellularia pseudoschizostoma* Hale, Smithson. Contr. Bot. 38: 28 (1978); *Chapsa pseudoschizostoma* (Hale) Sipman in Sipman et al., Phytotaxa 55: 46 (2012).

#### Pseudochapsa rhizophorae

(Kalb) Parnmen, Lücking & Lumbsch, comb. nov. [MycoBank MB 801531] Bas.: *Chapsa rhizophorae* Kalb, Herzogia 22: 28 (2009).

#### Pseudochapsa rivas-platae

(Kalb) Parnmen, Lücking & Lumbsch, comb. nov. [MycoBank MB 801532] Bas.: *Chapsa rivas-platae* Kalb, Herzogia 22: 29 (2009).

#### Pseudochapsa sipmanii

(Frisch & Kalb) Parnmen, Lücking & Lumbsch, comb. nov. [MycoBank MB 801533] Bas.: *Chapsa sipmanii* Frisch & Kalb, Biblioth. Lichenol. 99: 138 (2009).

#### Pseudochapsa subpatens

(Hale) Parnmen, Lücking & Lumbsch, comb. nov. [MycoBank MB 801534] Bas.: *Thelotrema subpatens* Hale, Bull. Br. Mus. Nat. Hist., Bot. 8: 269 (1981); *Chapsa subpatens* (Hale) Mangold in Mangold et al., Fl. Australia 57: 654 (2009).

#### Pseudotopeliopsis

Parnmen, Lücking & Lumbsch, gen. nov. [MycoBank MB 801543] Type species: *Pseudotopeliopsis laceratula* (Hale) Parnmen, Lücking & Lumbsch.

Differing from *Chapsa* s.str. in the densely corticate thallus and the *Topeliopsis*-like apothecia with striate excipulum filling the disc.

Thallus usually with dense cortex, rarely with loose cortex. Apothecia erumpent, rounded to irregular in outline; disc pore-like; margin fissured lobulate, in concentric layers covering the disc. Excipulum hyaline to brown. Ascospores septate to muriform, fusiform-ellipsoid to oblong-cylindrical, with slightly thickened septa and angular lumina (subdistoseptate), colorless to brown, I–. Secondary chemistry: no substances.

Etymology: Derived from “pseudo” (Greek, false) and the genus name *Topeliopsis*.

### New Combinations in *Pseudotopeliopsis*

#### Pseudotopeliopsis aggregate

(Hale) Parnmen, Lücking & Lumbsch, comb. nov. [MycoBank MB 801535] Bas.: *Phaeotrema aggregatum* Hale, Smithson. Contr. Bot. 16: 29 (1974); *Chapsa aggregata* (Hale) Sipman & Lücking in Rivas Plata et al., Lichenologist 42: 182 (2010).

#### Pseudotopeliopsis laceratula

(Müll. Arg.) Parnmen, Lücking & Lumbsch, comb. nov. [MycoBank MB 801536] Bas.: *Thelotrema laceratulum* Müll. Arg., Flora 70: 399 (1887); *Chapsa laceratula* (Müll. Arg.) Rivas Plata & Lücking in Rivas Plata et al., Lichenologist 42: 183 (2010).

#### Pseudotopeliopsis scabiocarpa

(Hale) Parnmen, Lücking & Lumbsch, comb. nov. [MycoBank MB 801537] Bas.: *Chapsa scabiocarpa* Rivas Plata & Lücking, Fung. Div. (in press).

#### Pseudotopeliopsis scabiomarginata

(Hale) Parnmen, Lücking & Lumbsch, comb. nov. [MycoBank MB 801538] Bas.: *Thelotrema scabiomarginatum* Hale, Bull. Br. Mus. Nat. Hist., Bot. 8: 269 (1981); *Chapsa scabiomarginata* (Hale) Rivas Plata & Lücking in Rivas Plata et al., Lichenologist 42: 183 (2010).

## Materials and Methods

### Taxon Sampling and Molecular Methods

We assembled a three-locus data set consisting of mtSSU rDNA, nuLSU rDNA, and the protein-coding genes *RPB2*. The taxon sampling contained 60 species focusing on the tribe Thelotremateae (Table 1). The outgroup taxa were chosen based on previous phylogenetic results [33]. New sequences were generated for this study using the Sigma REDExtract-N-Amp Plant PCR Kit (St. Louis, Missouri, SA) for DNA isolation following the manufacturer’s instructions, except that 40 µL of extraction buffer and 40 µL dilution buffer were used. DNA dilutions (5x) were used in PCR reactions of the genes coding for the nuLSU, mtSSU and RPB2, respectively.Primers for amplification were: (a) for nuLSU: AL2R [35], and nu-LSU-1125-3′ ( = LR6) [49], (b) for mtSSU: mr-SSU1 and Mr-SSU3R [50], and (c) for *RPB2*: fRPB2-7cF and fRPB2-11aR [51]. The cycle sequencing conditions were as follows: 96°C for 1 minute, followed by 25 cycles of 96°C for 10 seconds, 50°C for 5 seconds and 60°C for 4 minutes. Samples were precipitated and sequenced using Applied Biosystems 3730 DNA Analyzer (Foster City, California, U.S.A.). Sequence fragments obtained were assembled with SeqMan 4.03 (DNASTAR) and manually adjusted.

### Sequences Alignments and Phylogenetic Analyses

Alignments were done using Geneious Pro 5.5.2 [52]. Ambiguously aligned portions were removed manually. The single-locus and concatenated alignments were analyzed by maximum likelihood (ML) and a Bayesian approach (B/MCMC). To test for potential conflict, ML bootstrap analyses were performed on the individual data sets, and 75% bootstrap consensus trees were examined for conflict [2].

The ML analysis of the concatenated alignment was performed with the program RAxML-HPC2 (version 7.3.1) on XSEDE [53] using the default rapid hill-climbing algorithm. The model of nucleotide substitution chosen was GTRGAMMA. The data set was partitioned into five parts (mtSSU, nuLSU and each codon position of *RPB2*), so each gene partition was treated as an independent data set. Rapid bootstrap estimates were carried out for 1000 pseudoreplicates [54].

The B/MCMC analysis was conducted on the concatenated data set using MrBAYES 3.1.2 [55], with the same substitution model as in the ML analysis. A run with 10,000,000 generations, starting with a random tree and employing four simultaneous chains, was executed. No molecular clock was assumed. Heating of chains was set to 0.2. Posterior probabilities were approximated by sampling trees using a variant of Markov Chain Monte Carlo (MCMC) method. To avoid autocorrelation, only every 1000th tree was sampled. The first 4,000 generations were discarded as burn in. We used AWTY [56] to compare splits frequencies in the different runs and to plot cumulative split frequencies to ensure that stationarity was reached. Of the remaining19992 trees (9996 from each of the parallel runs) a majority rule consensus tree with average branch lengths was calculated using the sumt option of MrBAYES. Posterior probabilities were obtained for each clade. Clades with bootstrap support above 70% under ML and posterior probabilities ≥0.95 were considered as strongly supported. Phylogenetic trees were visualized using the program Treeview [57].

### Anatomical and Chemical Studies

Anatomical studies were conducted using standard light microscopy on hand-cut sections mounted in water. Secondary lichen substances were identified by thin-layer chromatography (TLC) and high performance thin-layer chromatography (HPTLC) according to standard methods [58], [59].

### Hypothesis Testing

Our phylogenetic analyses revealed that the genus *Chapsa* did not form a monophyletic group. Thus we tested whether our data are sufficient to reject monophyly of this genus. For the hypothesis testing, we used two different methods: (1) Shimodaira- Hasegawa (SH) test [60] and (2) expected likelihood weight (ELW) test [61]. The SH and ELW test were performed using Tree-PUZZLE v.5.2 [62] with the concatenated dataset, comparing the best tree agreeing with the null hypotheses, and the unconstrained ML tree. These trees were inferred in Tree-PUZZLE using the GTR+I+G nucleotide substitution model.

### Morphology-based Phylogenetic Binning

Since the molecular data set corresponding to the genus *Chapsa* included 21 species, but the entire genus considered here comprises 86 accepted species, molecular data were unavailable for 65 species or about 75% of all currently accepted species. In this case, the phylogenetic binning method provides a statistical approach for a predictive classification of species, by weighting the morphological characters based on their distribution on the phylogenetic tree of the sequenced species and then placing each additional species known from morphological characters only separately in the tree and testing alternative placements by means of bootstrapping [32]. The weighting can be applied using both an MP and an ML approach. In this case, we applied the binning method for three alternative solutions: a 2-genus, a 4-genus, and a 5-genus solution. We and used both MP and ML weighting, to generate six possible alternative classifications of *Chapsa* based on both molecular and morphological data: ML-2, MP-2, ML-4, MP-4, ML-5, and MP5.

### Multi-response Permutation Procedure

A multiresponse permutation procedure is a simple and effective tool to test for differences between groups of entities, in this case the groups obtained by the four alternative classifications obtained from the molecular phylogeny and subsequent binning method [63]. Since the morphological data matrix included only binary and ordered multistate characters, both Euclidean distances and linear correlation coefficients between each element within and between each group were computed. Within- and between group distances were then compared and statistical significance was tested by random data permutation using random shuffling of group partitions [63]. If within-group distances are smaller than expected by chance, it supports the recognition of a group as taxon, since such a result is evidence for partially independent phenotypic evolution. The analysis was performed in PC-Ord 5.03 [63]. For each individual character, we also employed Kruskal-Wallis ANOVA using each of the alternative clade solutions as grouping variable, to test whether the character discriminates between the resulting groups; this analysis was done in STATISTICA™ 6.0.

### Nomenclature

The electronic version of this article in Portable Document Format (PDF) in a work with an ISSN or ISBN will represent a published work according to the International Code of Nomenclature for algae, fungi, and plants, and hence the new names contained in the electronic publication of a PLOS ONE article are effectively published under that Code from the electronic edition alone, so there is no longer any need to provide printed copies.

In addition, new names contained in this work have been submitted to MycoBank from where they will be made available to the Global Names Index. The unique MycoBank number can be resolved and the associated information viewed through any standard web browser by appending the MycoBank number contained in this publication to the prefix http://www.mycobank.org/MB/. The online version of this work is archived and available from the following digital repositories: PubMed Central, LOCKSS.

## Supporting Information

Appendix S1 **Clade placement of taxa according to molecular phylogenetic analysis and phylogenetic binning according to the different classification solutions using 2, 4, or 5 clades under either ML or MP weighting.**
(DOC)

## References

[1] Hibbett DS, Binder M, Bischoff JF, Blackwell M, Cannon PF, et al (2007) A higher-level phylogenetic classification of the Fungi. Mycological Research 111: 509–547. 17572334 10.1016/j.mycres.2007.03.004

[2] Lutzoni F, Kauff F, Cox C, McLaughlin D, Celio G, et al (2004) Assembling the fungal tree of life: progress, classification, and evolution of subcellular traits. American Journal of Botany 91: 1446–1480. 21652303 10.3732/ajb.91.10.1446

[3] McLaughlin DJ, Hibbett DS, Lutzoni F, Spatafora JW, Vilgalys R (2009) The search for the fungal tree of life. Trends in Microbiology 17: 488–497. 19782570 10.1016/j.tim.2009.08.001

[4] Hillis DM (1987) Molecular versus morphological approaches to systematics. Annual Review of Ecology and Systematics 18: 23–42.

[5] Thomas RH, Hunt JA (1993) Phylogenetic relationships in *Drosophila*: a conflict between molecular and morphological data. Molecular Biology and Evolution 10: 362–374. 8487635 10.1093/oxfordjournals.molbev.a040008

[6] Wiens JJ, Hollingsworth BD (2000) War of the iguanas: Conflicting phylogenies, long branch attraction, and disparate rates of molecular and morphological evolution in iguanid lizards. Systematic Biology 49: 69–85. 10.1080/10635150050207447 12116477

[7] Lee MSY (2001) Uninformative characters and apparent conflict between molecules and morphology. Molecular Biology and Evolution 18: 676–680. 11264420 10.1093/oxfordjournals.molbev.a003848

[8] Wiens JJ (2004) The role of morphological data in phylogeny reconstruction. Systematic Biology 53: 653–661. 15371253 10.1080/10635150490472959

[9] Blanco O, Crespo A, Elix JA, Hawksworth DL, Lumbsch HT (2004) A molecular phylogeny and a new classification of parmelioid lichens containing *Xanthoparmelia*-type lichenan (Ascomycota: Lecanorales). Taxon 53: 959–975.

[10] Hibbett DS (2007) After the gold rush, or before the flood? Evolutionary morphology of mushroom-forming fungi (Agaricomycetes) in the early 21st century. Mycological Research 111: 1001–1018. 17964768 10.1016/j.mycres.2007.01.012

[11] Lumbsch HT, Huhndorf SM (2007) Whatever happened to the pyrenomycetes and loculoascomycetes? Mycological Research 111: 1064–1074. 18029164 10.1016/j.mycres.2007.04.004

[12] Moore J, Willmer P (1997) Convergent evolution in invertebrates. Biological Reviews 72: 1–60. 9116163 10.1017/s0006323196004926

[13] Stearns S, Hoekstra R (2005) Evolution: An Introduction. Oxford: Oxford University Press.

[14] Barton NH, Briggs DEG, Eisen JA, Goldstein DB, Patel NH (2007) Evolution. Cold Spring: Harbor Laboratory Press.

[15] Rivas Plata E, Lumbsch HT (2011) Parallel evolution and phenotypic divergence in lichenized fungi: a case study in the lichen-forming fungal family Graphidaceae (Ascomycota: Lecanoromycetes: Ostropales). Molecular Phylogenetics and Evolution 61: 45–63. 21605691 10.1016/j.ympev.2011.04.025

[16] Futuyama DJ (2005) Evolution. Sunderland: Sinauer Associates.

[17] Lumbsch HT, Huhndorf SM (2010) Myconet Volume 14. Part One. Outline of Ascomycota - 2009. Fieldiana (Life and Earth Sciences) 1: 1–42.

[18] Zalar P, de Hoog GS, Schroers HJ, Frank JM, Gunde-Cimerman N (2005) Taxonomy and phylogeny of the xerophilic genus Wallemia (Wallemiomycetes and Wallemiales, cl. et ord. nov.). Antonie Van Leeuwenhoek International Journal of General and Molecular Microbiology 87: 311–328. 10.1007/s10482-004-6783-x 15928984

[19] Vilnet AA, Milyutina IA, Konstantinova NA, Ignatov MS, Troitsky AV (2007) Phylogeny of the genus *Lophozia* (Dumort.) Dumort. s. str. inferred from nuclear and chloroplast sequences ITS1–2 and TRNL-F. Russian Journal of Genetics 43: 1306–1313. 18186195

[20] Fucikova K, Rada JC, Lewis LA (2011) The tangled taxonomic history of *Dictyococcus, Bracteacoccus* and *Pseudomuriella* (Chlorophyceae, Chlorophyta) and their distinction based on a phylogenetic perspective. Phycologia 50: 422–429.

[21] Fucikova K, Lewis LA (2011) Unravelling the taxonomic knot of *Bracteacoccus, Dictyococcus, Pseudomuriella,* and *Chromochloris* (Chlorophyceae, Chlorophyta): A case of cryptic genera. Journal of Phycology 47: S39–S39.

[22] Hebert PDN, Penton EH, Burns JM, Janzen DH, Hallwachs W (2004) Ten species in one: DNA barcoding reveals cryptic species in the neotropical skipper butterfly *Astraptes fulgerator* . Proceedings of the National Academy of Sciences of the United States of America 101: 14812–14817. 15465915 10.1073/pnas.0406166101 PMC522015

[23] Bickford D, Lohman DJ, Sodhi NS, Ng PKL, Meier R, et al (2007) Cryptic species as a window on diversity and conservation. Trends in Ecology & Evolution 22: 148–155. 17129636 10.1016/j.tree.2006.11.004

[24] Burns JM, Janzen DH, Hajibabaei M, Hallwachs W, Hebert PDN (2008) DNA barcodes and cryptic species of skipper butterflies in the genus *Perichares* in Area de Conservacion Guanacaste, Costa Rica. Proceedings of the National Academy of Sciences of the United States of America 105: 6350–6355. 18436645 10.1073/pnas.0712181105 PMC2359806

[25] Crespo A, Perez-Ortega S (2009) Cryptic species and species pairs in lichens: A discussion on the relationship between molecular phylogenies and morphological characters. Anales del Jardín Botánico de Madrid 66: 71–81.

[26] Seifert B (2009) Cryptic species in ants (Hymenoptera: Formicidae) revisited: we need a change in the alpha-taxonomic approach. Myrmecological News 12: 149–166.

[27] Trontelj P, Fiser C (2009) Cryptic species diversity should not be trivialised. Systematics and Biodiversity 7: 1–3.

[28] Crespo A, Lumbsch HT (2010) Cryptic species in lichen-forming fungi. IMA Fungus 1: 167–170. 22679576 10.5598/imafungus.2010.01.02.09 PMC3348775

[29] van Sickle J (1997) Using mean similarity dendrograms to evaluate classifications. Journal of Agricultural, Biological, and Environmental Statistics 2: 370–388.

[30] Mielke PW, Berry KJ (2001) Permutation Methods: A Distance Function Approach. Berlin: Springer Verlag.

[31] McCune B, Grace JB (2002) Analysis of Ecological Communities. Gleneden Beach, Oregon, USA: MjM Software Design.

[32] Berger SA, Stamatakis A, Lücking R (2011) Morphology-based phylogenetic binning of the lichen genera *Graphis* and *Allographa* (Ascomycota: Graphidaceae) using molecular site weight calibration. Taxon 60: 1450–1457.

[33] Rivas Plata E, Parnmen S, Staiger B, Mangold A, Frisch A, et al.. (2012) A molecular phylogeny of Graphidaceae (Ascomycota: Lecanoromycetes: Ostropales) including 437 species. MycoKeys in press.

[34] Rivas-Plata E, Lücking R, Lumbsch HT (2012) A new classification for the lichen family Graphidaceae s.lat. (Ascomycota: Lecanoromycetes: Ostropales). Fungal Diversity 52: 107–121.

[35] Mangold A, Martin MP, Lücking R, Lumbsch HT (2008) Molecular phylogeny suggests synonymy of Thelotremataceae within Graphidaceae (Ascomycota : Ostropales). Taxon 57: 476–486.

[36] Papong K, Corush J, Mangold A, Lucking R, Lumbsch HT (2009) Phylogenetic position of the foliicolous genus *Chroodiscus* (Ostropales, Ascomycota) inferred from nuclear and mitochondrial ribosomal DNA sequences. Fungal Diversity 38: 147–153.

[37] Frisch A, Kalb K, Grube M (2006) Contributions towards a new systematics of the lichen family Thelotremataceae. Bibliotheca Lichenologica 92: 1–539.

[38] Rivas Plata E, Lücking R, Sipman HJM, Mangold A, Lumbsch HT (2010) A world-wide key to the thelotremoid *Graphidaceae*, excluding the *Ocellularia*-*Myriotrema*-*Stegobolus* clade. Lichenologist 42: 139–185.

[39] Alexopoulos CJ, Mims CW, Blackwell M (1996) Introductory Mycology. New York: John Wiley & Sons.

[40] Jahns HM, Ott S (1994) Thallic mycelial and cytological characters in ascomycete systematics. In: Hawksworth DL, editor. Ascomycete Systematics Problems and Perspectives in the Nineties: NATO Advanced Science Institutes Series, Plenum Press, New York. 57–62.

[41] Kirk PM, Cannon PF, David JC, Stalpers JAe (2009) Ainsworth & Bisby’s Dictionary of the Fungi. 10th edition: CAB International, Wallingford, Oxon. 655 p.

[42] Lumbsch HT, Leavitt SD (2011) Goodbye morphology? A paradigm shift in the delimitation of species in lichenized fungi Fungal Diversity 50: 59–72.

[43] Mayr E (1963) Animal species and evolution. Cambridge, MA: Harvard University Press.

[44] Wang HY, Lumbsch HT, Guo SY, Huang MR, Wei JC (2010) Ascomycetes have faster evolutionary rates and larger species diversity than basidiomycetes. Science China, Life Sciences 53: 1163–1169. 20953937 10.1007/s11427-010-4063-8

[45] Funk DJ, Omland KE (2003) Species-level paraphyly and polyphyly: Frequency, causes, and consequences, with insights from animal mitochondrial DNA. Annual Review of Ecology Evolution and Systematics 34: 397–423.

[46] Rivas Plata E (2011) Historical biogeography, ecology and systematics of the family Graphidaceae (Lichenized Ascomycota: Ostropales). Chicago: University of Illinois at Chicago. 238 p.

[47] Ertz D, Lawrey JD, Sikaroodi M, Gillevet PM, Fischer E, et al (2008) A new lineage of lichenized basidiomycetes inferred from a two-gene phylogeny: The Lepidostromataceae with three species from the tropics. American Journal of Botany 95: 1548–1556. 21628162 10.3732/ajb.0800232

[48] Hodkinson BP, Uehling JK, Smith ME (2012) *Lepidostroma vilgalysii*, a new basidiolichen from the New World. Mycological Progress 11: 827–833.

[49] Vilgalys R, Hester M (1990) Rapid genetic identification and mapping of enzymatically amplified ribosomal DNA from several *Cryptococcus* species. Journal of Bacteriology 172: 4238–4246. 2376561 10.1128/jb.172.8.4238-4246.1990 PMC213247

[50] Zoller S, Scheidegger C, Sperisen C (1999) PCR primers for the amplification of mitochondrial small subunit ribosomal DNA of lichen-forming ascomycetes. Lichenologist 31: 511–516.

[51] Liu YJ, Whelen S, Hall BD (1999) Phylogenetic relationships among ascomycetes: evidence from an RNA polymerase II subunit. Molecular Biology and Evolution 16: 1799–1808. 10605121 10.1093/oxfordjournals.molbev.a026092

[52] Drummond A, Ashton B, Buxton S, Cheung M, Cooper A, et al. (2011) Geneious v5.4. Available from http://www.geneious.com/.

[53] Stamatakis A (2006) RAxML-VI-HPC: Maximum Likelihood-based Phylogenetic Analyses with Thousands of Taxa and Mixed Models. Bioinformatics 22: 2688–2690. 16928733 10.1093/bioinformatics/btl446

[54] Stamatakis A, Hoover P, Rougemont J (2008) A Rapid Bootstrap Algorithm for the RAxML Web Servers. Systematic Biology 57: 758–771. 18853362 10.1080/10635150802429642

[55] Huelsenbeck JP, Ronquist F (2001) MRBAYES: Bayesian inference of phylogenetic trees. Bioinformatics 17: 754–755. 11524383 10.1093/bioinformatics/17.8.754

[56] Nylander JAA, Wilgenbusch JC, Warren DL, Swofford DL (2007) AWTY (Are We There Yet?): a system for graphical exploration of MCMC convergence in Bayesian phylogenetics. Bioinformatics 24: 581–583. 17766271 10.1093/bioinformatics/btm388

[57] Page RDM (1996) Treeview: an application to display phylogenetic trees on personal computers. Computer Applied Biosciences 12: 357–358. 10.1093/bioinformatics/12.4.357 8902363

[58] Arup U, Ekman S, Lindblom L, Mattsson J-E (1993) High performance thin layer chromatography (HPTLC), an improved technique for screening lichen substances. Lichenologist 25: 61–71.

[59] Orange A, James PW, White FJ (2001) Microchemical Methods for the Identification of Lichens: British Lichen Society. 101 p.

[60] Shimodaira H, Hasegawa M (2001) CONSEL: for assessing the confidence of phylogenetic tree selection. Bioinformatics 17: 1246–1247. 11751242 10.1093/bioinformatics/17.12.1246

[61] Strimmer K, Rambaut A (2002) Inferring confidence sets of possibly misspecified gene trees. Proceedings of the Royal Society of London Series B, Biological Sciences 269: 137–142. 11798428 10.1098/rspb.2001.1862 PMC1690879

[62] Schmidt HA, Strimmer K, Vingron M, von Haeseler A (2002) TREE-PUZZLE: maximum likelihood phylogenetic analysis using quartets and parallel computing. Bioinformatics 18: 502–504. 11934758 10.1093/bioinformatics/18.3.502

[63] McCune B, Mefford MJ (1999) PC-ORD. Multivariate Analysis of Ecological Data, Version 4.0. Gleneden Beach, OR: MjM Software Design. 237 p.

