# Supplementary material for: Phylogenetic Classification at Generic Level in the Absence of Distinct Phylogenetic Patterns of Phenotypical Variation: A Case Study in Graphidaceae (Ascomycota)
Source: PLoS One. 2012 Dec 12;7(12):e51392. doi: 10.1371/journal.pone.0051392 (PMC3520900; doi:10.1371/journal.pone.0051392)
Supplement: Appendix S1 — Clade placement of taxa according to molecular phylogenetic analysis and phylogenetic binning according to the different classification solutions using 2, 4, or 5 clades under either ML or MP weighting. (DOC) [file pone.0051392.s001.doc]

**Appendix S1.** Clade placement of taxa according to molecular phylogenetic analysis and phylogenetic binning according to the different classification solutions using 2, 4, or 5 clades under either ML or MP weighting.

| **Species** | **Authors** | **ML_2** | **MP_2** | **ML_4** | **MP_4** | **ML_5** | **MP_5** | **Genus** |
| --- | --- | --- | --- | --- | --- | --- | --- | --- |
| *alstrupii* | Frisch | Clade II | Clade II | Subclade IIc | Subclade IIc | Subclade IIc | Subclade IIc | *Astrochapsa* |
| *amazonica* | Kalb | Clade II | Clade II | Subclade IIc | Subclade IIc | Subclade IIc | Subclade IIc | *Astrochapsa* |
| *astroidea* | (Berk. & Broome) Cáceres & Lücking | Clade II | Clade II | Subclade IIc | Subclade IIc | Subclade IIc | Subclade IIc | *Astrochapsa* |
| *calathiformis* | (Vain.) Lumbsch & Papong | Clade II | Clade II | Subclade IIc | Subclade IIc | Subclade IIc | Subclade IIc | *Astrochapsa* |
| *graphidioides* | Kalb | Clade II | Clade II | Subclade IIc | Subclade IIc | Subclade IIc | Subclade IIc | *Astrochapsa* |
| *lassae* | Mangold | Clade II | Clade II | Subclade IIc | Subclade IIc | Subclade IIc | Subclade IIc | *Astrochapsa* |
| *magnifica* | (Berk. & Broome) Rivas Plata & Mangold | Clade II | Clade II | Subclade IIc | Subclade IIc | Subclade IIc | Subclade IIc | *Astrochapsa* |
| *mastersonii* | Rivas Plata, Lumbsch & Lücking | Clade II | Clade II | Subclade IIc | Subclade IIc | Subclade IIc | Subclade IIc | *Astrochapsa* |
| *megaphlyctidioides* | Mangold | Clade II | Clade II | Subclade IIc | Subclade IIc | Subclade IIc | Subclade IIc | *Astrochapsa* |
| *meridensis* | (Kalb & Frisch) Lücking, Lumbsch & Rivas Plata | Clade II | Clade II | Subclade IIc | Subclade IIc | Subclade IIc | Subclade IIc | *Astrochapsa* |
| *platycarpella* | (Vain.) Frisch | Clade II | Clade II | Subclade IIc | Subclade IIc | Subclade IIc | Subclade IIc | *Astrochapsa* |
| *pseudophlyctis* | (Nyl.) Frisch | Clade II | Clade II | Subclade IIc | Subclade IIc | Subclade IIc | Subclade IIc | *Astrochapsa* |
| *pulvereodiscus* | (Hale) Rivas Plata & Mangold | Clade II | Clade II | Subclade IIc | Subclade IIc | Subclade IIc | Subclade IIc | *Astrochapsa* |
| *recurva* | (G. Salisb.) Frisch | Clade II | Clade II | Subclade IIc | Subclade IIc | Subclade IIc | Subclade IIc | *Astrochapsa* |
| *stellata* | (Hale) Sipman | Clade II | Clade II | Subclade IIc | Subclade IIc | Subclade IIc | Subclade IIc | *Astrochapsa* |
| *waasii* | (Hale) Sipman & Lücking | Clade II | Clade II | Subclade IIc | Subclade IIc | Subclade IIc | Subclade IIc | *Astrochapsa* |
| *wolseleyana* | (Hale) Sipman & Lücking | Clade II | Clade II | Subclade IIc | Subclade IIc | Subclade IIc | Subclade IIc | *Astrochapsa* |
| *zahlbruckneri* | (Redinger) Frisch | Clade II | Clade II | Subclade IIc | Subclade IIc | Subclade IIc | Subclade IIc | *Astrochapsa* |
| *alborosella* | (Nyl.) Frisch | Clade I | Clade I | Clade I | Clade I | Subclade Ib | Subclade Ib | *Chapsa s.str.* |
| *boninensis* | (Tat. Matsumoto) Rivas Plata & Mangold | Clade I | Clade I | Clade I | Clade I | Subclade Ia | Subclade Ia | *Chapsa s.str.* |
| *chionostoma* | (Nyl.) Rivas Plata & Mangold | outside | outside | Clade I | Clade I | Subclade Ia | Subclade Ia | *Chapsa s.str.* |
| *cinchonarum* | (Fée) Frisch | Clade I | Clade I | Clade I | Clade I | Subclade Ia | Subclade Ia | *Chapsa s.str.* |
| *defecta* | Lücking | Clade I | Clade I | Clade I | Clade I | Subclade Ib | Subclade Ib | *Chapsa s.str.* |
| *defectosorediata* | Lücking | Clade I | Clade I | Clade I | Clade I | Subclade Ib | Subclade Ib | *Chapsa s.str.* |
| *diploschistoides* | (Zahlbr.) Frisch | Clade I | Clade I | Clade I | Clade I | Subclade Ia | Subclade Ia | *Chapsa s.str.* |
| *eitenii* | (Hale) Frisch | Clade I | Clade I | Clade I | Clade I | Subclade Ia | Subclade Ia | *Chapsa s.str.* |
| *halei* | Mangold | Clade I | outside | Clade I | Clade I | Subclade Ib | Subclade Ia | *Chapsa s.str.* |
| *hiata* | (Hale) Sipman | Clade I | Clade I | Clade I | Clade I | Subclade Ia | Subclade Ia | *Chapsa s.str.* |
| *imperfecta* | (Hale) Rivas Plata & Lücking | Clade I | Clade I | Clade I | Clade I | Subclade Ib | Subclade Ib | *Chapsa s.str.* |
| *indica* | A. Massal. | Clade I | Clade I | Clade I | Clade I | Subclade Ia | Subclade Ia | *Chapsa s.str.* |
| *laemensis* | (Homchantara & Coppins) Lumbsch & Papong | Clade I | Clade I | Clade I | Clade I | Subclade Ib | Subclade Ib | *Chapsa s.str.* |
| *leprocarpa* | (Nyl.) Frisch | Clade I | Clade I | Clade I | Clade I | Subclade Ia | Subclade Ia | *Chapsa s.str.* |
| *meghalayensis* | (Patw. & Nagarkar) Lumbsch & Divakar | Clade I | Clade I | Clade I | Clade I | Subclade Ib | Subclade Ib | *Chapsa s.str.* |
| *niveocarpa* | Mangold | Clade I | Clade I | Clade I | Clade I | Subclade Ia | Subclade Ia | *Chapsa s.str.* |
| *patens* | (Nyl.) Frisch | Clade I | Clade I | Clade I | Clade I | Subclade Ia | Subclade Ia | *Chapsa s.str.* |
| *pulchra* | (Müll. Arg.) Mangold | Clade I | Clade I | Clade I | Clade I | Subclade Ia | Subclade Ia | *Chapsa s.str.* |
| *pulchrella* | Wijeyaratne, Lücking & Lumbsch | Clade I | Clade I | Clade I | Clade I | Subclade Ia | Subclade Ia | *Chapsa s.str.* |
| *sublilacina* | (Ellis & Everhart) Sipman & Lücking | Clade I | Clade I | Clade I | Clade I | Subclade Ib | Subclade Ib | *Chapsa s.str.* |
| *sublilacina var. cyanea* | Lücking | Clade I | Clade I | Clade I | Clade I | Subclade Ib | Subclade Ib | *Chapsa s.str.* |
| *thallotrema* | Lücking & N. Salazar | Clade I | Clade I | Clade I | Clade I | Subclade Ia | Subclade Ia | *Chapsa s.str.* |
| *tibellii* | Mangold | Clade I | Clade I | Clade I | Clade I | Subclade Ib | Subclade Ib | *Chapsa s.str.* |
| *asteliae* | (Kantvilas & Vezda) Mangold | Clade II | Clade II | Subclade IIb | Subclade IIa | Subclade IIb | Subclade IIa | *provisionally in Chapsa s.lat.* |
| *discoides* | (Stirt.) Lücking | Clade II | Clade I | Subclade IIc | Clade I | Subclade IIc | Subclade Ia | *provisionally in Chapsa s.lat.* |
| *elabens* | (Müll. Arg.) Rivas Plata & Mangold | Clade I | Clade II | Clade I | Subclade IIa | Subclade Ia | Subclade IIa | *provisionally in Chapsa s.lat.* |
| *granulifera* | Frisch & Kalb | Clade II | Clade I | Subclade IIa | Clade I | Subclade IIa | Subclade Ib | *provisionally in Chapsa s.lat.* |
| *grossomarginata* | (Tat. Matsumoto) Mangold | Clade II | Clade I | Subclade IIc | Clade I | Subclade IIc | Subclade Ia | *provisionally in Chapsa s.lat.* |
| *lordhowensis* | Mangold | Clade I | Clade II | Clade I | Subclade IIa | Subclade Ib | Subclade IIa | *provisionally in Chapsa s.lat.* |
| *microspora* | Kalb | outside | Clade II | Clade I | Subclade IIb | Subclade Ia | Subclade IIb | *provisionally in Chapsa s.lat.* |
| *minor* | (Kantvilas & Vezda) Mangold & Lumbsch | Clade II | Clade II | Subclade IIc | Subclade IIa | Subclade IIc | Subclade IIa | *provisionally in Chapsa s.lat.* |
| *mirabilis* | (Zahlbr.) Lücking | Clade II | Clade I | Subclade IIc | Clade I | Subclade IIc | Subclade Ia | *provisionally in Chapsa s.lat.* |
| *neei* | (Hale) Sipman, Mangold & Lücking | Clade II | Clade II | Subclade IIa | Subclade IIb | Subclade IIa | Subclade IIb | *provisionally in Chapsa s.lat.* |
| *pallidella* | Kalb | Clade II | Clade II | Subclade IIc | Subclade IIa | Subclade IIc | Subclade IIa | *provisionally in Chapsa s.lat.* |
| *paralbida* | (Riddle) Lücking | Clade II | Clade II | Subclade IIa | Subclade IIc | Subclade IIa | Subclade IIc | *provisionally in Chapsa s.lat.* |
| *perdissuta* | Sipman & Lücking | Clade II | Clade II | Subclade IIb | Subclade IIc | Subclade IIb | Subclade IIc | *provisionally in Chapsa s.lat.* |
| *rubropulveracea* | Hale ex Mangold, Lücking & Lumbsch | Clade II | Clade I | Subclade IIa | Clade I | Subclade IIa | Subclade Ib | *provisionally in Chapsa s.lat.* |
| *sorediata* | Kalb | Clade II | Clade I | Subclade IIa | Clade I | Subclade IIa | Subclade Ib | *provisionally in Chapsa s.lat.* |
| *subsorediata* | Rivas Plata & Lücking | Clade II | Clade I | Subclade IIa | Clade I | Subclade IIa | Subclade Ib | *provisionally in Chapsa s.lat.* |
| *wijeyaratniana* | Weerakon, Lumbsch & Lücking | Clade II | Clade I | Subclade IIa | Clade I | Subclade IIa | Subclade Ib | *provisionally in Chapsa s.lat.* |
| *albomaculata* | (Sipman) Sipman & Lücking | Clade II | Clade II | Subclade IIa | Subclade IIa | Subclade IIa | Subclade IIa | *Pseudochapsa* |
| *crispata* | (Müll. Arg.) Rivas Plata & Mangold | Clade II | Clade II | Subclade IIa | Subclade IIa | Subclade IIa | Subclade IIa | *Pseudochapsa* |
| *dilatata* | (Müll. Arg.) Kalb | Clade II | Clade II | Subclade IIa | Subclade IIa | Subclade IIa | Subclade IIa | *Pseudochapsa* |
| *esslingeri* | (Hale) Sipman | Clade II | Clade II | Subclade IIa | Subclade IIa | Subclade IIa | Subclade IIa | *Pseudochapsa* |
| *hypoconstictica* | Rivas Plata & Lücking | Clade II | Clade II | Subclade IIa | Subclade IIa | Subclade IIa | Subclade IIa | *Pseudochapsa* |
| *isidiifera* | Frisch & Kalb | Clade II | Clade II | Subclade IIa | Subclade IIa | Subclade IIa | Subclade IIa | *Pseudochapsa* |
| *kalbii* | Frisch | Clade II | Clade II | Subclade IIa | Subclade IIa | Subclade IIa | Subclade IIa | *Pseudochapsa* |
| *lueckingii* | Kalb | Clade II | Clade II | Subclade IIa | Subclade IIa | Subclade IIa | Subclade IIa | *Pseudochapsa* |
| *phlyctidea* | (Nyl.) Lücking | Clade II | Clade II | Subclade IIa | Subclade IIa | Subclade IIa | Subclade IIa | *Pseudochapsa* |
| *phlyctidioides* | (Müll. Arg.) Mangold | Clade II | Clade II | Subclade IIa | Subclade IIa | Subclade IIa | Subclade IIa | *Pseudochapsa* |
| *pseudoexanthismocarpa* | (Patw. & C. R. Kulk.) Rivas Plata & Lücking | Clade II | Clade II | Subclade IIa | Subclade IIa | Subclade IIa | Subclade IIa | *Pseudochapsa* |
| *pseudoschizostoma* | (Hale) Sipman | Clade II | Clade II | Subclade IIa | Subclade IIa | Subclade IIa | Subclade IIa | *Pseudochapsa* |
| *rhizophorae* | Kalb | Clade II | Clade II | Subclade IIa | Subclade IIa | Subclade IIa | Subclade IIa | *Pseudochapsa* |
| *rivas-platae* | Kalb & Lücking | Clade II | Clade II | Subclade IIa | Subclade IIa | Subclade IIa | Subclade IIa | *Pseudochapsa* |
| *sipmanii* | Frisch & Kalb | Clade II | Clade II | Subclade IIa | Subclade IIa | Subclade IIa | Subclade IIa | *Pseudochapsa* |
| *subpatens* | (Hale) Mangold | Clade II | Clade II | Subclade IIa | Subclade IIa | Subclade IIa | Subclade IIa | *Pseudochapsa* |
| *aggregata* | (Hale) Sipman & Lücking | Clade II | Clade II | Subclade IIa | Subclade IIb | Subclade IIa | Subclade IIb | *Pseudotopeliopsis* |
| *laceratula* | (Müll. Arg.) Rivas Plata & Lücking | Clade II | Clade II | Subclade IIb | Subclade IIb | Subclade IIb | Subclade IIb | *Pseudotopeliopsis* |
| *scabiocarpa* | Rivas Plata & Lücking | Clade II | Clade II | Subclade IIb | Subclade IIb | Subclade IIb | Subclade IIb | *Pseudotopeliopsis* |
| *scabiomarginata* | (Hale) Rivas Plata & Lücking | Clade II | Clade II | Subclade IIc | Subclade IIb | Subclade IIc | Subclade IIb | *Pseudotopeliopsis* |
| *albida* | (Nyl.) Lücking & Sipman | Clade II | Clade II | Subclade IIb | Subclade IIb | Subclade IIb | Subclade IIb | *requires sequencing* |
| *dissuta* | (Hale) Mangold | Clade II | Clade II | Subclade IIa | Subclade IIa | Subclade IIa | Subclade IIa | *requires sequencing* |
| *farinosa* | Lücking & Sipman | Clade II | Clade II | Subclade IIc | Subclade IIc | Subclade IIc | Subclade IIc | *requires sequencing* |
| *lamellifera* | (Kantvilas & Vezda) Mangold | Clade II | Clade II | Subclade IIb | Subclade IIb | Subclade IIb | Subclade IIb | *requires sequencing* |
| *leprocarpoides* | (Hale) Cáceres & Lücking | Clade II | Clade II | Subclade IIb | Subclade IIb | Subclade IIb | Subclade IIb | *requires sequencing* |
| *dilatatoides* | unpublished | Clade II | Clade II | Subclade IIc | Subclade IIc | Subclade IIc | Subclade IIc | *unpublished* |
| *lucens* | unpublished | Clade II | Clade II | Subclade IIa | Subclade IIa | Subclade IIa | Subclade IIa | *unpublished* |
| *subdilatata* | unpublished | Clade II | Clade II | Subclade IIa | Subclade IIa | Subclade IIa | Subclade IIa | *unpublished* |
